# Association of Term Labor Induction vs Expectant Management With Child Academic Outcomes

**DOI:** 10.1001/jamanetworkopen.2020.2503

**Published:** 2020-04-10

**Authors:** Erika F. Werner, Lauren E. Schlichting, William A. Grobman, Samara Viner-Brown, Melissa Clark, Patrick M. Vivier

**Affiliations:** 1 Hassenfeld Child Health Innovation Institute, Brown University Providence, Rhode Island; 2 Brown University School of Public Health, Providence, Rhode Island; 3 Department of Obstetrics and Gynecology, Northwestern University, Chicago, Illinois; 4 Rhode Island Department of Health, Providence

## Abstract

**Importance:**

Although labor induction at 39 weeks of gestation has been shown to reduce the number of cesarean deliveries, compared with expectant management, without increasing neonatal morbidity in nulliparous, low-risk women, the association between induction at 39 weeks and longer-term childhood cognitive outcomes is not certain.

**Objective:**

To evaluate educational outcomes of children born by induction at 39 or 40 weeks compared with those whose mothers were expectantly managed beyond those weeks.

**Design, Setting, and Participants:**

This statewide cohort study was conducted in Rhode Island. The participants included children of nulliparous women who were born at 39 weeks of gestation or later and then completed third-grade math and reading tests during the 2014 to 2017 academic year. Data analysis was performed from July 2019 to October 2019.

**Exposures:**

Induction of labor compared with expectant management.

**Main Outcomes and Measures:**

Third-grade math and reading test scores and proficiency (based on achievement level) among children born after induction in the 39th or 40th week were compared with scores for those who remained in utero beyond that same gestational week. The hypothesis was that induction in the 39th or 40th week would not be associated with differences in math or reading scores or proficiency compared with expectant management past the 39th or 40th week of gestation.

**Results:**

Of the 6393 children meeting the inclusion criteria (mean [SD] age, 8.00 [0.22] years; 3208 boys [50.2%]; 376 [5.8%] black; 1280 [22.0%] Hispanic), 455 were delivered by induction in the 39th week and 610 were delivered by induction in the 40th week. There were no differences in mean math or reading test scores or in the frequency of math or reading proficiency between children delivered by induction at 39 or 40 weeks compared with those whose mothers were expectantly managed (overall mean [SD] math score, 744 [33]; overall mean [SD] reading score, 743 [38]; 2945 children [46%] achieved proficiency in math and 2833 [44%] achieved proficiency in reading). After adjusting for plausible confounders (race/ethnicity, maternal education, hypertension, diabetes, and socioeconomic status), induction continued to be associated with similar proficiency in math and reading compared with expectant management. For children born by induction at 39 weeks, the adjusted relative risks were 1.07 (95% CI, 0.97-1.18) for math proficiency and 0.98 (95% CI, 0.88-1.08) for reading proficiency. For children born by induction at 40 weeks, the adjusted relative risks were 0.97 (95% CI, 0.88-1.08) for math proficiency and 0.98 (95% CI, 0.89-1.08) for reading proficiency.

**Conclusions and Relevance:**

These findings suggest that the offspring of nulliparous women for whom labor is induced at 39 or 40 weeks have similar third-grade educational outcomes compared with the offspring of mothers who underwent expectant management past those gestational ages.

## Key Points

**Question:**

Does academic achievement differ in children delivered by induction at term compared with those whose mothers were managed expectantly?

**Findings:**

In this cohort study, there were no differences in third-grade reading and math scores among children delivered by induction at 39 or 40 weeks compared with those whose mothers were expectantly managed past those gestational ages.

**Meaning:**

These findings suggest that labor induction at term does not appear to be associated with poorer test scores when those children are in third grade.

## Teaser

This cohort study evaluates the educational outcomes of children born by induction at 39 or 40 weeks of gestation compared with those whose mothers were expectantly managed past those gestational ages.

## Introduction

In August 2018, the results of the ARRIVE trial were published.^1^ In this multicenter trial, low-risk, nulliparous women were randomized to either undergo labor induction or expectant management at 39 weeks of gestation. The study demonstrated a reduced risk of cesarean delivery (18.6% vs 22.2%; relative risk [RR], 0.84; 95% CI, 0.76-0.93) and hypertensive disorders of pregnancy (9.1% vs 14.1%; RR, 0.64; 95% CI, 0.56-0.74) in the group of women assigned to induction compared with those assigned to expectant management.^1^ Furthermore, induction at 39 weeks of gestation compared with expectant management past 39 weeks was not associated with an increased risk of any neonatal complication. The results of this trial have led many clinicians to begin offering elective induction to low-risk nulliparous women at 39 weeks.

Prior studies^2,3,4,5,6,7,8^ that examined the association between gestational age at delivery and educational outcomes usually found a benefit to delivery, in the absence of complications, no earlier than 39 weeks. Some researchers^8^ have even suggested that optimal education outcomes occur when delivery occurs in the late-term period (≥41 weeks). However, those studies used the week of delivery as the primary exposure, which is not a comparison that provides useful information in terms of prospective clinical decision-making. Specifically, because standard obstetrical care has been to await labor until at least 41 weeks, with earlier delivery initiated only if complications (eg, hypertensive disorders of pregnancy) arise, women who reach later gestational ages are systematically more likely to be without complications and, correspondingly, their children are more likely to be in better health. Thus, for example, the correct comparison in trying to understand whether it is better for children to be delivered at 39 weeks of gestation compared with a later gestation is to compare those who are delivered at 39 weeks with all those who are delivered thereafter.

Therefore, we sought to use statewide third-grade math and reading assessment scores and to compare test scores of children who were born by induction at 39 or 40 weeks with scores of those whose mothers were expectantly managed past those weeks of gestation. We hypothesized that the offspring of nulliparous women for whom labor was induced at a given term gestational age would have similar math and reading scores compared with children born to women expectantly managed past the same gestational week.

## Methods

This cohort study was approved by the Rhode Island Department of Health human subjects institutional review board, which granted a consent waiver because all identified information remained at the state level. This study follows the Strengthening the Reporting of Observational Studies in Epidemiology (STROBE) reporting guideline.

The Rhode Island Department of Education provided third-grade data obtained between 2014 and 2017 from the Partnership for Assessment of Readiness for College and Careers statewide test of mathematics and English language arts and literacy. Beginning in third grade, the examination is administered near the end of the academic year, and all students enrolled in public schools are expected to participate unless they qualify for an alternative assessment or receive an exemption. Birth certificate data for births between 2005 and 2008 were also obtained from the Rhode Island Department of Health. Using probabilistic matching, birth certificates and records from KIDSNET, Rhode Island’s integrated child health information system, were matched using characteristics of both the child (date of birth, sex, first name, and last name) and the mother (date of birth, medical record number, and town of residence). These matched records were then directly merged with education records. Children with both birth certificate and education data comprise the study population.

For this study, we further limited the population to nulliparous women with a singleton pregnancy who were still pregnant at 39 weeks of gestation because we were most interested in the school-aged outcomes of children in the ARRIVE trial^1^ (Figure). The ARRIVE trial was limited to nulliparous women, and so we did the same in this study. Children for whom a major congenital anomaly (eg, chromosomal anomalies, microcephalus, spina bifida, or gastroschisis) was listed on the birth certificate were excluded. For children who repeated third grade, data for their first test attempt was retained and later test scores were excluded.

**Figure.**
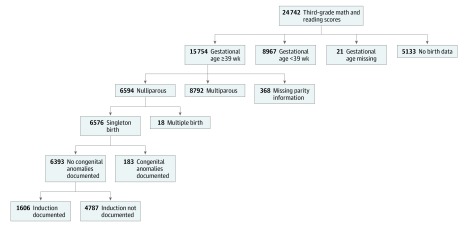
Study Population Flow Diagram

Within this study population, we compared children who were born after induction at 39 or 40 weeks of gestation with children whose mothers were expectantly managed past that week of gestation. The gestational age of delivery was based on the best clinical estimate as entered on the birth certificate by the delivering practitioner. The birth certificate only provides week of delivery, not day of delivery (eg, 39 weeks, not 39 weeks and 3 days). Induction was determined by the intervention category on the 1989 US Standard Certificate of Live Birth (which was in use in Rhode Island during 2005-2008), for which the delivering clinician could select none, stimulation (augmentation), or induction. If induction was selected on the birth certificate, the delivery was assumed to result from labor induction.

The primary outcome was third-grade math and reading scores on the Partnership for Assessment of Readiness for College and Careers examination from 2014 to 2017. Math and reading were evaluated by separate tests, and each score was evaluated both continuously as a scale score (range, 650-850) and as a binary variable using performance levels to assess competency. Performance levels are calculated by the Partnership for Assessment of Readiness for College and Careers administrators on the basis of a range of overall scale scores for the assessment and are used to report student proficiency. There are 5 performance levels with higher levels indicating better performance. Children at levels 4 (score, 750-784) and 5 (score, 785-850) have demonstrated readiness for the next grade level and are classified as proficient in the subject area. Children at levels 1 (score, 650-699), 2 (score, 700-724), and 3 (score, 725-749) have not met expectations and are classified as nonproficient.

Maternal, pregnancy, and child characteristics that, according to prior literature, were considered to be potential confounders between the exposure and outcomes of interest in this study were evaluated as covariates. Maternal information derived from birth certificates included age, education level, smoking status, and medical comorbidities, such as diabetes and hypertension. Data on mode of delivery and birth weight were also available from the birth certificate. With regard to the child, data on race/ethnicity, city of residence in third grade (core city vs noncore area), and participation in the National School Lunch Program were provided by the Rhode Island Department of Education. Core cities were defined as cities with 25% or more children living in poverty.

### Statistical Analysis

We estimated bivariable associations between math and reading test scores and induction compared with expectant management at a given gestation week. Standard deviations were reported for continuous variables where the mean was calculated. Interquartile ranges were reported when the median was calculated. Fisher exact tests were used where participant counts were fewer than 20, and χ^2^ tests were used for categorical variables. All tests were 2-sided.

Multivariable logistic regression was used to determine the RR of improved test scores given expectant management in a specific week after adjusting for potential confounders. Covariates were considered for inclusion in the multivariable model if there was biological plausibility and if any bivariable χ^2^ *P* values were less than .05. Covariates were also examined for collinearity, and if colinearity was statistically significant (defined as *P* < .05), the variable that had more biological plausibility to affect the outcome of interest was adjusted for in the final model. Analyses were conducted using SAS statistical software version 9.4 (SAS Institute). Data analysis was performed from July 2019 to October 2019.

## Results

Ultimately, 6393 children met the study criteria for inclusion (mean [SD] age, 8.00 [0.22] years; 3208 boys [50.2%]; 376 black [5.8%]; 1280 Hispanic [22.0%]). Their mothers’ mean (SD) age at delivery was 25 (6) years, 1048 mothers (18.0%) had less than a high school education at the time of delivery, 310 mothers (4.1%) had hypertension, and 180 mothers (3.2%) had diabetes. Of the 24 742 children with complete (both math and reading) 2014 to 2017 third grade education records, 5133 could not be successfully linked to birth certificates, 8967 children were born before 39 weeks’ gestation, 389 had missing gestational age or parity data, 8792 were born to multiparous mothers, and 201 were excluded because of congenital anomalies or multiple gestation (Figure). In the final cohort of 6393 study participants, 455 were born by induction at 39 weeks, and 3812 were expectantly managed past 39 weeks; 610 were born by induction at 40 weeks, and 1149 were expectantly managed past 40 weeks. For the entire cohort, the mean (SD) math score was 744 (33) and the mean reading score was 743 (38), with 2945 children (46%) achieving proficiency (a score of 4 or 5) in math and 2833 (44%) achieving proficiency in reading.

Induction at both 39 and 40 weeks was not associated with any difference in third-grade math or reading scores compared with expectant management (Table 1). Math and reading scores were nearly identical in the induction group (39 weeks: mean [SD] math score, 744.4 [34.0], and mean [SD] reading score, 742.7 [37.3]; 40 weeks: mean [SD] math score, 744.8 [32.2], and mean [SD] reading score, 744.7 [38.1]) compared with the expectant management group (39 weeks: mean [SD] math score, 744.7 [33.7], and mean [SD] reading score, 743.2 [38.7]; 40 weeks: mean [SD] math score, 744.9 [33.1], and mean [SD] reading score, 743.4 [38.8]). Proficiency levels were also nearly identical for the 2 groups. Of the children born by induction at 39 weeks, 206 (45.3%) were proficient in math and 188 (41.3%) were proficient in reading; of the children whose mothers were expectantly managed past the 39th week, 1736 (45.5%) were proficient in math and 1688 (44.3%) were proficient in reading. Of the children born by induction at 40 weeks, 275 (45.1%) were proficient in math and 269 (44.1%) were proficient in reading; of the children whose mothers were expectantly managed past the 40th week, 532 (46.3%) were proficient in math and 512 (44.6%) were proficient in reading.

**Table 1.** Third Grade Math and Reading Scores Among Children Born by Induction Compared With Those Expectantly Managed at Term

Variable	Induction in 39th wk (n = 455)	Expectant management past 39th wk (n = 3812)	*P* value	Induction in 40th wk (n = 610)	Expectant management past 40th wk (n = 1149)	*P* value
Math						
Score, mean (SD)	744.4 (34.0)	744.7 (33.7)	.87	744.8 (32.2)	744.9 (33.1)	.98
Proficiency, participants, No. (%)	206 (45.3)	1736 (45.5)	.91	275 (45.1)	532 (46.3)	.63
Reading						
Score, mean (SD)	742.7 (37.3)	743.2 (38.7)	.79	744.7 (38.1)	743.4 (38.8)	.51
Proficiency, participants, No. (%)	188 (41.3)	1688 (44.3)	.23	269 (44.1)	512 (44.6)	.85

When potential covariates were examined, there was no difference in terms of maternal age, mode of delivery, or maternal smoking status among women whose labor was induced compared with those expectantly managed (Table 2). Women whose labor was induced in each given week were more likely to have hypertension (39 weeks, 92 women [20.2%] vs 127 women [3.3%]; 40 weeks, 69 women [11.3%] vs 22 women [1.9%]) or diabetes (39 weeks, 55 women [12.1%] vs 78 women [2.1%]; 40 weeks, 34 women [5.6%] vs 13 women [1.1%]) and to have infants with lower birth weights (39 weeks, mean [SD], 3384 [451] g vs 3543 [424] g; 40 weeks, mean [SD], 3523 [411] g vs 3633 [422] g) compared with women who were expectantly managed. Child’s race/ethnicity (Hispanic, 103 children [16.9%] vs 238 children [20.7%]; non-Hispanic white, 443 children [72.6%] vs 745 children [64.8%]), maternal education (less than high school, 81 women [13.8%] vs 203 women [18.1%]), and measures of socioeconomic status, including receiving lunch subsidies (free lunch, 223 children [36.6%] vs 489 children [42.6%]) or living in a core city (149 children [24.4%] vs 343 children [29.9%]), differed between those born by induction compared with those expectantly managed in the 40th week but not in the 39th week (Table 2).

**Table 2.** Characteristics of Children Born by Induction vs Those Expectantly Managed and Their Mothers

Characteristic	Participants, No. (%)					
	Induction in 39th wk	Expectant management past 39th wk	*P* value	Induction in 40th wk	Expectant management past 40th wk	*P* value
Maternal age, mean (SD), y	25.3 (6.1)	25.5 (6.2)	.53	25.9 (6.00)	25.7 (6.3)	.56
Child’s race/ethnicity						
Hispanic	98 (21.5)	841 (22.0)	.18	103 (16.9)	238 (20.7)	.01
Non-Hispanic						
White	282 (62.0)	2465 (64.7)		443 (72.6)	745 (64.8)	
Black	30 (6.6)	235 (6.2)		30 (4.9)	81 (7.1)	
Other	45 (9.9)	271 (7.1)		34 (5.6)	85 (7.4)	
Maternal education						
Less than high school	80 (18.0)	684 (18.5)	.12	81 (13.8)	203 (18.1)	.03
High school diploma	162 (36.6)	1177 (31.8)		208 (35.4)	386 (34.3)	
Any college	201 (45.4)	1838 (49.7)		298 (50.8)	535 (47.6)	
Lunch subsidy						
Free	195 (42.9)	1597 (41.9)	.59^a^	223 (36.6)	489 (42.6)	.03
Reduced	16 (3.5)	175 (4.6)		25 (4.1)	56 (4.9)	
Core city	129 (28.4)	1123 (29.5)	.62	149 (24.4)	343 (29.9)	.02
Material comorbidities						
Hypertension	92 (20.2)	127 (3.3)	<.001	69 (11.3)	22 (1.9)	<.001
Diabetes	55 (12.1)	78 (2.1)	<.001	34 (5.6)	13 (1.1)	<.001^a^
Smoking	43 (9.8)	353 (9.6)	.91	64 (10.8)	118 (10.7)	.96
Mode of delivery						
Cesarean	158 (34.7)	1277 (33.5)	.34	236 (38.7)	473 (41.2)	.53
Vaginal	261 (57.4)	2148 (56.4)		314 (51.5)	573 (50.0)	
Forceps or vacuum	36 (7.9)	383 (10.1)		60 (9.8)	101 (8.8)	
Birth weight, mean (SD), g	3384 (451)	3543 (424)	<.001	3523 (411)	3633 (422)	<.001

^a^ Fisher exact test.

After adjusting for confounders, induction was not associated with differences in math or reading proficiency compared with expectant management during either period. For children born by induction at 39 weeks, the adjusted RRs were 1.07 (95% CI, 0.97-1.18) for math proficiency and 0.98 (95% CI, 0.88-1.08) for reading proficiency. For children born by induction at 40 weeks, the adjusted RRs were 0.97 (95% CI, 0.88-1.08) for math proficiency and 0.98 (95% CI, 0.89-1.08) for reading proficiency (Table 3). Core city residence at birth was not included in the multivariable model because it was associated with lunch subsidy, another measure of poverty.

**Table 3.** Adjusted RRs of Math or Reading Proficiency Comparing Induction With Expectant Management During a Given Week of Gestation at Term

Variable	Proficient, Adjusted RR (95% CI)			
	39 wk		40 wk	
	Math	Reading	Math	Reading
Group				
Induction	1.07 (0.97-1.18)	0.98 (0.88-1.08)	0.97 (0.88-1.08)	0.98 (0.89-1.08)
Expectant management	1 [Reference]	1 [Reference]	1 [Reference]	1 [Reference]
Maternal hypertension				
Yes	0.85 (0.73-1.00)	1.03 (0.90-1.18)	0.79 (0.60-1.05)	1.00 (0.81-1.24)
No	1 [Reference]	1 [Reference]	1 [Reference]	1 [Reference]
Maternal diabetes				
Yes	0.75 (0.59-0.94)	0.99 (0.83-1.19)	0.75 (0.51-1.11)	0.92 (0.67-1.28)
No	1 [Reference]	1 [Reference]	1 [Reference]	1 [Reference]
Maternal education				
Less than high school	0.66 (0.57-0.76)	0.62 (0.54-0.73)	0.74 (0.60-0.92)	0.68 (0.54-0.85)
High school diploma	0.79 (0.73-0.85)	0.76 (0.70-0.83)	0.81 (0.72-0.92)	0.72 (0.63-0.82)
Any college	1 [Reference]	1 [Reference]	1 [Reference]	1 [Reference]
Lunch subsidy				
Free	0.61 (0.55-0.67)	0.60 (0.54-0.67)	0.65 (0.56-0.75)	0.61 (0.52-0.72)
Reduced	0.83 (0.70-0.99)	0.91 (0.77-1.07)	0.78 (0.58-1.04)	0.90 (0.70-1.16)
No subsidy	1 [Reference]	1 [Reference]	1 [Reference]	1 [Reference]
Child race/ethnicity				
Hispanic	0.86 (0.77-0.95)	0.73 (0.65-0.82)	0.82 (0.69-0.98)	0.73 (0.60-0.88)
Non-Hispanic				
White	1 [Reference]	1 [Reference]	1 [Reference]	1 [Reference]
Black	0.68 (0.56-0.84)	0.72 (0.59-0.88)	0.64 (0.46-0.89)	0.74 (0.55-0.99)
Other	0.97 (0.85-1.09)	0.93 (0.82-1.06)	1.03 (0.85-1.25)	0.93 (0.75-1.17)
Child sex				
Male	0.96 (0.91-1.02)	0.75 (0.71-0.80)	0.95 (0.87-1.04)	0.75 (0.67-0.82)
Female	1 [Reference]	1 [Reference]	1 [Reference]	1 [Reference]

Abbreviation: RR, relative risk.

## Discussion

With the publication of the ARRIVE trial, there will very likely be a greater proportion of nulliparous women whose children are delivered at 39 or 40 weeks of gestation after labor induction. Although observational and trial data show that short-term outcomes of children born at these gestational ages are similar to or better than those of children born later,^1,9,10^ it is also vital that the long-term outcomes of such a change are explored. To this end, we demonstrated that in a diverse population of children born to nulliparous women, labor induction at 39 weeks or 40 weeks was not associated with differences in third-grade math and reading scores, or in the frequency of math and reading proficiency, compared with expectant management.

Our study results differ from those of the only other large US study addressing educational outcomes of term compared with late-term delivery. Figlio et al^8^ performed a cross-sectional study of singleton children born in Florida between 1994 and 2002 who were educated in the Florida public schools. They compared Florida Comprehensive Assessment Test scores at ages 8 to 15 years between children born at term (39 or 40 weeks) with those born late term (≥41 weeks) and found that late-term infants scored 0.7% of an SD (95% CI, 0.1%-1.3%; *P* = .02) higher than did full-term infants, were 2.8% more likely to be gifted (95% CI, 0.4%-5.2%; *P* = .02), and were 3.1% less likely to have poor cognitive outcomes (95% CI, 0.0%-6.1%; *P* = .05) compared with full-term infants.^8^ These results likely differ from our results because of methodological differences. Figlio et^8^ al used cross-sectional design and compared all children born at 39 or 40 weeks with those born at 41 weeks of gestation or later. Thus, children who were born spontaneously in a given week were included with those who were born by induction for medical or elective reason. Such a comparison does not provide insight into the actual decision a woman and a practitioner must make in a clinical setting (ie, whether to deliver at a given time or wait beyond that time). That insight is provided by comparing women for whom labor was induced at a given gestational age with those who continued past that gestational age as we did.

### Limitations

Although our study question is very relevant to potential changes in obstetric patterns over the next decade, the study does have limitations. Most importantly, although our study focused on inductions, we did not have the ability to differentiate elective low-risk inductions from medically indicated inductions. Nevertheless, it should be noted that medically indicated inductions are likely associated with worse perinatal and childhood outcomes, and this potential bias drives the comparison away from the null and favors expectant management. Yet, even with this bias, we saw no differences between the groups. Also, the data included are from a single state. Although this state’s population is ethnically, racially, and socioeconomically diverse, differences between state education policies may still limit the generalizability of our findings. Our study results are further limited by the retrospective nature of the data, especially because much of the data are derived from birth certificates, leading to potential misclassification bias. Furthermore, the education outcomes are limited to third-grade scores because that is when all students in Rhode Island are tested. Ideally, we would have annual math and reading assessments for this population. In addition, the study required linking educational data to birth certificate data. Therefore, any child born outside the state, or who was born in the state but then moved out of the state, was excluded, allowing for overrepresentation of less-mobile families.

## Conclusions

This a large study that addresses a clinically relevant question by comparing school-age outcomes associated with induction at full term compared with expectant management. Our results, which showed no difference in school-age outcomes with induction compared with expectant management at 39 or 40 weeks, should reassure women and health care practitioners as they consider the options for timing of full-term delivery.
